# Molecular and immunological characterization of *Hyalomma dromedarii* and *Hyalomma excavatum* (Acari: Ixodidae) vectors of Q fever in camels

**DOI:** 10.14202/vetworld.2018.1109-1119

**Published:** 2018-08-12

**Authors:** Hend H. A. M. Abdullah, Eman E. El-Shanawany, Sobhy Abdel-Shafy, Hala A. A. Abou-Zeina, Eman H. Abdel-Rahman

**Affiliations:** Department of Parasitology and Animal Diseases, Veterinary Research Division, National Research Centre, Dokki, Giza, Egypt

**Keywords:** 16S ribosomal DNA, *Coxiella burnetii*, cytochrome oxidase subunit-1, hard ticks, phylogeny, polymerase chain reaction, sequence, sodium dodecyl sulfate-polyacrylamide gel electrophoresis, western blot

## Abstract

**Background and Aim:**

Q fever *Coxiella burnetii* is a worldwide zoonotic disease, and *C. burnetii* was detected in mammals and ticks. Ticks play an important role in the spread of *C. burnetii* in the environment. Therefore, the aims of this study were to detect Q fever *C. burnetii* in camels and ixodid ticks by molecular tools and identification of *Hyalomma dromedarii* and *Hyalomma excavatum* using molecular and immunological assays.

**Materials and Methods:**

A total of 113 blood samples from camels and 190 adult ticks were investigated for the infection with *C. burnetii* by polymerase chain reaction (PCR) and sequencing the targeting IS30A spacer. The two tick species *H. dromedarii* and *H. excavatum* were characterized molecularly by PCR and sequencing of 16S ribosomal RNA (16S rRNA) and cytochrome oxidase subunit-1 (*CO1*) genes and immunologically by sodium dodecyl sulfate-polyacrylamide gel electrophoresis (SDS-PAGE) and western blot.

**Results:**

A total of 52 camels (46%) were positive for Q fever infection. Only 10 adult ticks of *H. dromedarii* were infected with *C. burnetii*. The IS30A sequence was around 200 bp in length for *C. burnetii* in *H. dromedarii* ticks with a similarity of 99% when compared with reference data in GenBank records. The length of 16S rDNA and *CO1* was 440 and 850 bp, respectively, for both *H. dromedarii* and *H. excavatum*. The phylogenetic status of *H. dromedarii* was distant from that of *H. excavatum*. SDS-PAGE revealed seven different bands in the adult antigens of either *H. dromedarii* or *H. excavatum* with molecular weights ranged from 132.9 to 17.7 KDa. In western blot analyses, the sera obtained from either infested camel by *H. dromedarii* or infested cattle by *H. excavatum* recognized four immunogenic bands (100.7, 49.7, 43.9, and 39.6 kDa) in *H. dromedarii* antigen. However, the infested camel sera identified two immunogenic bands (117 and 61.4 kDa) in *H. excavatum* antigen. Furthermore, the sera collected from cattle infested by *H. excavatum* recognized three immunogenic bands (61.4, 47.3, and 35 kDa) in *H. excavatum* antigen.

**Conclusion:**

Molecular analyses indicated that both camels and ticks could be sources for infection of animals and humans with Q fever. Furthermore, the molecular analyses are more accurate tools for discriminating *H. dromedarii* and *H. excavatum* than immunological tools.

## Introduction

Q fever is a worldwide zoonotic disease caused by an obligate Gram-negative bacterium *Coxiella burnetii* [1]. In 1935, the first identified outbreak of the disease happened among Austrian abattoir workers [2]. Domestic animals such as cattle, sheep, and goats are known reservoirs of *C. burnetii* [3]. Antibodies to Q fever were detected in camels without any clinical signs [4,5]. The infection in animals is usually subclinical, but abortion and reduction in reproductive efficiency are the most critical investigation of such disease [6]. In human, the disease causes acute flu-like illness, hepatitis, pneumonia, and chronic endocarditis [7,8]. Recently, the genus *Coxiella* is closely related to the order Legionellales (Gammaproteobacteria) [9]. *C. burnetii* and *Coxiella cheraxi* (a pathogen of crayfishes [10]) are the only identified pathogens in such genus. Moreover, many *Coxiella*-like organisms were detected in the invertebrate species, especially ticks [11,12], depending on 16S rRNA gene sequencing as a universal DNA barcoding marker [13]. These organisms were closely related to *C. burnetii* but genetically distinct [14]. In addition, many of *C. burnetii* were isolated from animals and human during Q fever outbreaks [15-17] and in more than 40 tick species in different countries [18]. It multiplies in the tick-middle gut, resulting in high numbers of viable organisms that are excreted with feces, saliva, and coxal fluid. Ticks can transmit *C. burnetii* transstadially and transovarially to their offspring [19]. Ticks play an important role in the spread of *C. burnetii* in the environment, especially wildlife, because of the high concentration of this bacteria in tick feces [20,21].

The camel tick *Hyalomma dromedarii* Koch, 1844 (Acari: Ixodidae), prefers the camel as the primary host of the adult stage. It also infests other domestic animals such as cattle, sheep, goats, and equines. Larvae and nymphs feed on birds and small burrowing animals as rodents, but the nymphs can infest large animals like the adults. It commonly behaves as a two-host tick and appears throughout the year. It is found wherever camels live and it distributes mainly in North Africa, Mauritania, Middle East, India, Mongolia, Tibet, Ethiopia, Eretria, Kenya, and Uganda [22,23]. It is the most *Hyalomma* spp. parasitizing camels in Egypt [24-26]. It has a vital role in the transmission of emerging and reemerging diseases as *Theileria* [27,28], *Rickettsia* [29,30], *Francisella* [31], Q fever [32], and viruses [33].

The tick *Hyalomma excavatum* Koch, 1844 (Acari: Ixodidae), has a wide range of animal hosts as cattle, camels, equines, sheep, goats, and dogs and it can attack humans. It feeds as a two-host or three-host tick according to the availability of hosts. Larvae and nymphs feed on small vertebrates, birds, and human. It is found throughout the year with a reduced number in winter [34,35]. It is distributed mainly in North Africa including Egypt, Sudan, Ethiopia, Eritrea, Iran, Turkey, Italy, and Greece [36]. Furthermore, *H. excavatum* plays a role in the transmission of protozoan diseases as babesiosis and theileriosis [37-39], bacterial diseases as rickettsiosis [30], and viral diseases as Crimean Congo hemorrhagic fever [40].

Moreover, the identification of ticks depends on the morphological characteristics of the adult stages that are easily observed by light microscope. It is difficult to identify the ticks depending on the morphological features in three cases. The first case is the female that is laying eggs because the structure of its genital opening is not clear enough to distinguish the tick species. The second case is the damaged ticks, especially important taxonomic parts, such as mouthparts, scutum, and genital opening. The third case is the immature stages which need more advanced tools to identify ticks at the species level [41,42]. Scanning electron microscope was used to describe the immature stages of *H. dromedarii*, *H. excavatum*, and *Hyalomma marginatum* [43]. He found that the nymphal stage was well differentiated between the three species, while the larval stage of each *H. dromedarii* and *H. excavatum* is very close to each other. This finding led to a search for other tools to confirm the identification of ticks in such case.

In Egypt, there is not enough available data about Q fever in camels linked with their tick vectors. Therefore, the aims of this study were to detect Q fever *C. burnetii* in camels and ixodid ticks using PCR and sequencing of the targeting IS30A spacer and to evaluate 16S ribosomal RNA (16S rRNA) and cytochrome oxidase subunit-1 (*CO1*) genes using PCR and sequencing as molecular methods and sodium dodecyl sulfate-polyacrylamide gel electrophoresis (SDS-PAGE) and western blot as immunological methods for characterization of the two tick species *H. dromedarii* and *H. excavatum*.

## Materials and Methods

### Ethical approval

This study was approved to be carried out by the Ethics Committee at the National Research Centre in Egypt.

### Animals

A total of 113 camels were examined for the presence of ticks on different parts of their bodies according to Abdullah *et al*. [25] from Cairo, Giza, and Sinai in Egypt during the period from June to August 2017. Blood samples with ethylenediaminetetraacetic acid (EDTA) (5 ml each animal) were collected from jugular veins and were stored at −20°C for molecular purposes.

### Ticks

A total of 268 ticks were collected from camels from Cairo, Giza, and Sinai in Egypt during the period mentioned above. Ticks were removed from camels using curved forceps into plastic tubes. Then, the tubes were covered with cloths and secured by rubber bands. Ticks were brought alive to the laboratory for further identification and investigation. The adult ticks were identified by the key of Estrada-Pena *et al*. [36]. Then, the ticks were stored at −20°C until DNA was extracted for molecular procedures. *H. dromedarii* and *H. excavatum* were chosen for the molecular and immunological investigations.

### Molecular techniques

#### DNA extraction

DNA was extracted from blood using GF-1 Tissue Blood Combi DNA Extraction Kit (SNF, Vivantis, Malaysia) according to the manufacturer’s instructions. Furthermore, DNA was extracted and purified from adult ticks individually after dissection a tick into two halves. The high salt concentration protocol was used for DNA extraction [44]. The NanoDrop 2000c (Thermo Scientific) was used for the measurement of the purity and concentration of DNA that was stored at −20°C until used in PCR procedures.

#### PCR protocol

The primers used in this study are presented in Table-1 [21,45,46]. The PCR reaction was performed in 25 µl total volume under aseptic condition. Each PCR mixture contained 25-50 ng/µl genomic DNA, 10 pM/µl of each primer, 12.5 µl of 2× PCR Master Mix solution (i-Taq) PCR master mix (1× buffer, 2.5 mM dNTP, 1× gel loading buffer, and 4 mM MgCl_2_; iNtRon Biotechnology, Korea), and 9 µl nuclease-free water (Qiagen) to complete the total volume of the reaction. All amplifications were performed in BIO-RAD Thermal Cycler (BIO-RAD, Singapore) utilizing the following cycling profile, for the two DNA markers one cycle at 94°C for 5 min (initial denaturation). The PCR protocol of *CO1* and 16S rRNA genes was amplified 35 cycles of denaturation at 94°C for 1 min, annealing at 45°C for 30 s and elongation at 72°C for 1 min, and the final elongation at 72°C for 10 min [45,46]. While, the IS30A protocol included 40 cycles of denaturation at 94°C for 30 s, annealing at 52°C for 30 s and elongation at 72°C for 1 min, and then the final elongation at 72°C for 5 min [21]. A reagent blank was run as control simultaneously with every PCR. The PCR products were inspected by electrophoresis in 1.5% agarose gel in tris-borate-EDTA (TBE) buffer (45 mM TBE, 1 mM EDTA, pH 8.3, Sigma-Aldrich) and stained with ethidium bromide (Sigma-Aldrich). A gene ruler 100 bp plus DNA ladder (Thermo Scientific, California, USA) was used with each gel. Gel photos were analyzed by Lab Image software (BioRad) [29].

**Table-1:** Primers utilized in PCR amplification and sequencing of genes.

DNA marker	5’ Primers Sequences3’	Amplified fragments	References
IS30-A IS30A-F IS30A-R	5’ CGCTGACCTACAGAAATATGTCC3’ 5’ GGGGTAAGTAAATAATACCTTCTGG3’	196-166 bp	[21]
*CO1* *CO1*-F *CO1*-R	5’ GGAACAATATATTTAATTTTTGG3’ 5’ ATCTATCCCTACTGTAAATATATG3’	732-820 bp	[45]
16S rDNA 16S-F 16S-R	5’ TTAAATTGCTGTRGTATT3’ 5’ CCGGTCTGAACTCASAWC3’	455 bp	[46]

*CO1*=Cytochrome oxidase subunit1

#### Sequencing of the PCR products

PCR products were purified for sequencing using ExoSAP-IT PCR Product Cleanup Kit (Affymetrix, Ohio, USA) according to manufacturer’s recommendation. Sequencing reactions were performed in an MJ Research PTC-225 Peltier Thermal Cycler using an ABI PRISM^®^ BigDye™ terminator cycle sequencing kits with AmpliTaq^®^ DNA polymerase (FS enzyme; Applied Biosystems), following the protocols supplied by the manufacturer.

#### Phylogenetic analyses and tree construction

Amplified sequences of each fragment were aligned using Blastn program of NCBI (http://www.ncbi.nlm.nih.gov/BLAST/) for sequence homology searches against GenBank database. Multiple sequence alignments for evolutionary relationships between obtained sequences and another reference in GenBank were inferred using the ClustalW 1.8^®^ program [47], after modification of sequence length by BioEdit sequence alignment editor (v. 7.0.9.0). Two phylogenetic trees were constructed with the neighbor-joining method [48] and the unweighted pair group method with arithmetic mean (UPGMA) [49]. The evolutionary distances were calculated by the maximum composite likelihood method [50]. Branches corresponding to partitions reproduced in <50% of bootstrap replicates were collapsed. Percentage of replicate trees in which the associated taxa clustered together in the bootstrap test (500 replicates) is shown next to the branches [51,52]. Phylogenetic analyses were conducted in MEGA4 [53].

### Immunological techniques

#### Antigen preparation

Extraction of antigens was done in an ice bath. Adult female ticks of *H. dromedarii* and *H. excavatum* were triturated in a sterile glass mortar with 0.15 mol/L PBS (pH 7.2). The homogenate was sonicated and centrifuged at 13,000 rpm for 1 h in a cooling centrifuge. The supernatant crude antigen was aliquot then preserved at −20 until used. The protein concentration of extract was estimated by the method of Lowry *et al*. [54].

#### SDS-PAGE

About 30 µg of *H. dromedarii* or *H. excavatum* adult crude antigen was mixed with reducing sample buffer and loaded separately in 10% SDS-PAGE [55]. Gels were silver stained according to the method of Wray *et al*. [56]. Molecular weight prestained protein ladder (Fermentas) ranged from 10 to 260 kDa was electrophoresed in the same gel for comparative purposes.

#### Western blotting

Preparation of buffers, samples, and the transfer procedure was carried out according to the method described by Towbin *et al*. [57]. The resolved proteins were transferred to 0.2 μm nitrocellulose membranes (Sigma, USA). Membranes were subsequently blocked with 5% skimmed milk in Tris-buffered saline with Tween^®^ 20 (TBST) buffer consisting of Tris, 125 mM NaCl, and 0.1% Tween 20 for 1 h at room temperature followed by incubation with primary antibodies (camel naturally infested by *H. dromedarii* and cattle naturally infested by *H. excavatum*) diluted at 1:100 in 0.01 M Tris-buffered saline, pH 7.5, containing 0.5% bovine serum albumin, overnight at 4°C. Excess antibodies were removed by extensive washing in TBST, and blots were then reprobed with horseradish peroxidase (HRP)-conjugated protein A 1/2,000 dilution (Sigma, USA). Membranes were then washed extensively with TBST, and primary antibody binding was visualized with 4-chloro-1-naphthol as a substrate.

## Results

### Molecular detection of Q fever

**Figure-1:**
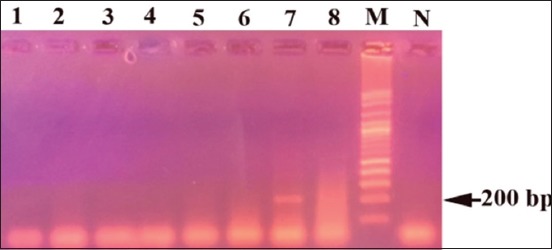
Agarose gel electrophoresis of polymerase chain reaction products obtained from *H. dromedarii* using IS30A spacer: (M) 1 kb DNA ladder, (N) negative control, lanes 7 and 8 represent positive tick samples and lanes 1-6 represent negative tick samples.

**Figure-2:**
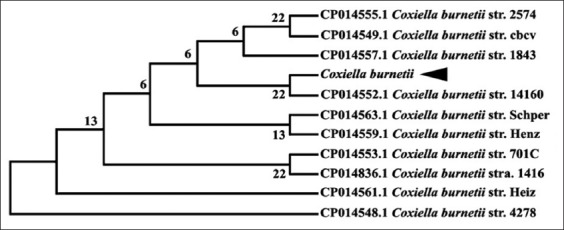
Phylogenetic tree of the IS30A sequences for *Coxiella burnetii* recorded in GenBank, including *C. burnetii* obtained from *Hyalomma dromedarii* in this study.

A total of 113 blood samples from camels and 190 adult ticks were investigated for the infection with *C. burnetii* by PCR using IS30A spacer. The overall prevalence of Q fever in camels was 46%, including 36 (67%) and 16 (27.1%) from Cairo and Giza, respectively. Only 10 adult ticks of *H. dromedarii* were infected with *C. burnetii*. The infected ticks were found at Giza Governorate and represented 5.3% of the total ticks (Table-2). The obtained IS30A sequence was around 200 bp in length for *C. burnetii* in *H. dromedarii* ticks with a similarity of 99% when compared with other records of *C. burnetii* in GenBank (Figures-1 and 2).

**Table-2:** The prevalence of *C. burnetii* in camels and their ticks by PCR using IS30A spacer.

Governorates	Camels No.	Positive number of camels with Q fever (prevalence %)*	Ticks		Positive number of ticks with Q fever (prevalence %)

			Species	No.	
Cairo	53	36 (67.9)	*H. dromedarii*	79	0
			*H. excavatum*	4	0
			*H. rufipes*	4	0
			*H. marginatum*	2	0
Giza	59	16 (27.1)	*H. dromedarii*	45	10 (5.3)
			*H. excavatum*	1	0
Sinai	1	0 (0)	*H. dromedarii*	53	0
			*H. marginatum*	2	0
Total	113	52 (46.01)	Total	190	10/190 (5.3)

**χ^2^=7.692 (p=0.006). *H. dromedarii=Hyalomma dromedarii, H. excavatum=Hyalomma excavatum, H. marginatum=Hyalomma marginatum, H. rufipes=Hyalomma rufipes, C. burnetii=Coxiella burnetii,* PCR=Polymerase chain reaction

### Molecular characterization of tick species

**Figure-3:**
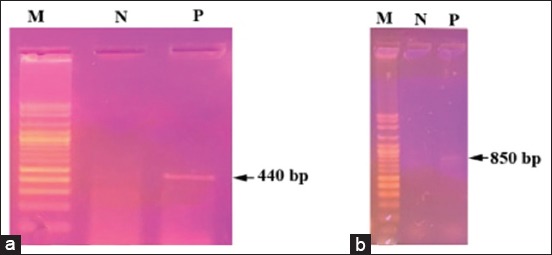
Agarose gel electrophoresis of polymerase chain reaction products obtained from *Hyalomma dromedarii* and *Hyalomma excavatum* using two genes: (a) 16S ribosomal RNA gene reveals 440 bp, (b) cytochrome oxidase subunit-1 gene, reveals 850 bp, (M) 1 kb DNA ladder, (N) negative control.

**Figure-4:**
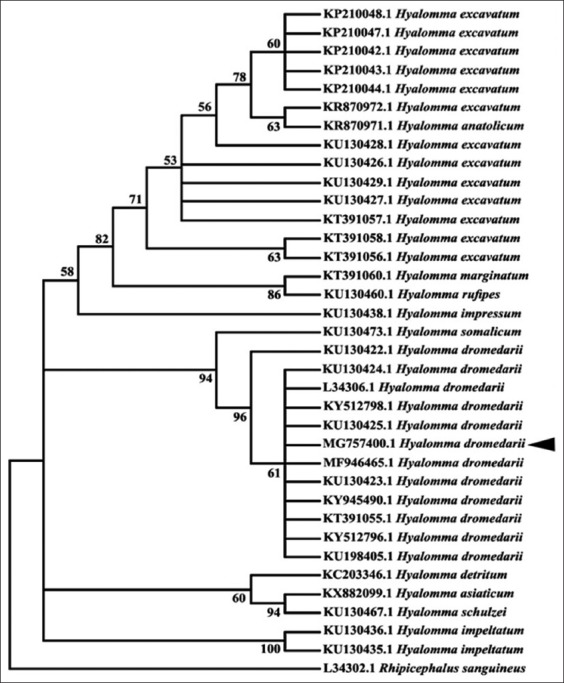
Phylogenetic tree of the 16S rDNA sequences for *Hyalomma* spp. recorded in GenBank, including *Hyalomma dromedarii* obtained in this study with accession number MG757400.

**Figure-5:**
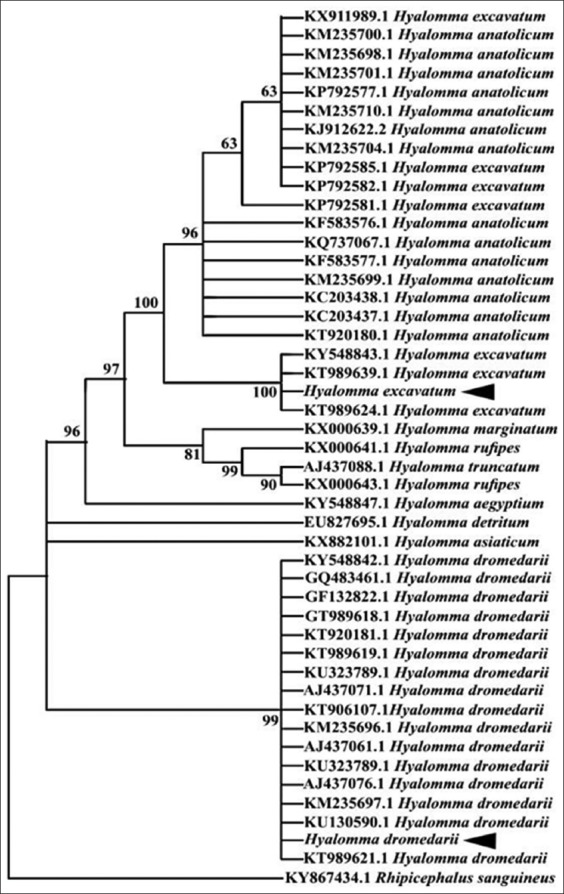
Phylogenetic tree of the cytochrome oxidase subunit-1 sequences for *Hyalomma* spp. recorded in GenBank, including *Hyalomma dromedarii* and *Hyalomma excavatum* obtained in this study.

The partial 16S rRNA gene revealed band around 440 bp in length for both *H. dromedarii* and *H. excavatum* (Figure-3a). The band around 850 bp in length was appeared with partial *CO1* gene for the two *Hyalomma* species (Figure-3b). The similarity of *H. dromedarii* for the obtained 16S rDNA sequence to the same tick species recorded in Genbank was 99%, while the PCR product of *H. excavatum* 16S rRNA gene failed to produce a good sequence. The phylogenetic trees of 16S rRNA and *CO1* genes for *H. dromedarii* confirmed that these species locate in the same glade with others *H. dromedarii* recorded in GenBank (Figures-4 and 5). The phylogenetic tree of *CO1* gene for *H. excavatum* was also in a separate glade with others *H. excavatum* previously recorded in GenBank (Figure-5).

### Immunological characterization

**Figure-6:**
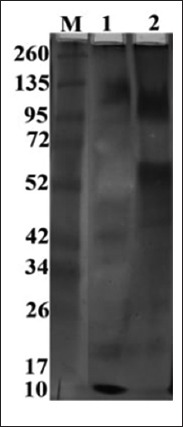
10% sodium dodecyl sulfate-polyacrylamide gel electrophoresis of the isolated *Hyalomma dromedarii* and *Hyalomma excavatum* total adult crude proteins. Lane 2: whole *H. dromedarii*, Lane 3: *H. excavatum*. Tick protein bands were compared to ferment as wide range standards to estimate molecular weight.

SDS-PAGE and silver staining analysis indicate unique protein profiles for each crude tick species (*H. dromedarii* and *H. excavatum*). The electrophoretic pattern of crude *H. dormedarii* adult antigen was released seven bands of different molecular weights 132.9, 100.7, 74, 49.7, 43.9, 39.6, and 17.7 kDa, while the *H. excavatum* crude adult antigen resolved seven bands differ from those of *H. dromedarii*, wherever they were 117.0, 61.4, 52.1, 47.3, 35, 29, and 18.9 kDa (Figure-6).

**Figure-7:**
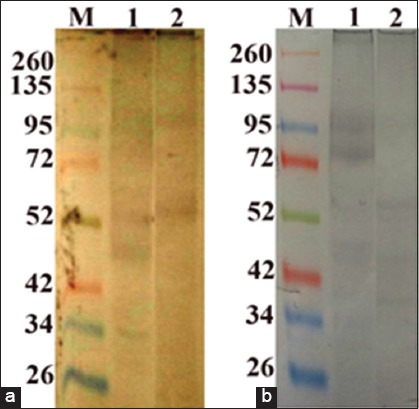
Immunoblot analysis of total protein adult crude extracts from different tick species. The samples from the following species were analyzed: (1) *Hyalomma dromedarii* and (2) *Hyalomma excavatum*. The blots were probed with camel sera naturally infested by *H. dromedarii* (a), and cattle sera naturally infested by *H. excavatum* (b). (M) ferment as wide range prestained molecular weight standard.

The reactivity of naturally infested camel sera against the whole *H. dromedarii* adult tick was determined through four major protein bands with molecular weight 100.7, 49.7, 43.9, and 39.6 kDa, while the reactivity of it with cattle sera naturally infested by *H. excavatum* was at 100.7, 74, 49.7, and 43.9 kDa. Western blot analysis of tick *H. excavatum* crude adult extract with naturally infested cattle sera observed that there are reactions at three antigenic sites with 61.4, 47.3, and 35 kDa, while their reaction with camel naturally infested sera showed two bands only at 117 and 61.4 kDa. This result demonstrates the cross-reactivity between *H. dromedarii* and *H. excavatum* (Figure-7).

## Discussion

It is known that the ixodid ticks are vectors for many protozoan, bacterial, and viral pathogens. The capability of disease transmission differs from tick species to another. Most of the diseases transmitted by ticks do not restrict to animals but extend to humans [58]. In particular, bacterial diseases such as *Rickettsia* spp., *Borrelia* spp., and *C. burnetii* cause health problems for animals and humans [59,60]. Studies on *C. burnetii* (Q fever) in Egypt are limited, especially in camels and tick vectors. Therefore, the first step of this study was conducted to investigate the presence of Q fever in camels and related ticks using PCR and sequencing analyses.

Furthermore, PCR and sequencing were used to achieve the second step that related to the examination of the two genes 16S rRNA and *CO1* for molecular characterization of the camel tick *H. dromedarii* and the tick *H. excavatum*. The selection of 16S rRNA and *CO1* genes was based on the previous studies [25,45,46] whose stated that these DNA markers are more reliable in discriminating species of ticks. Whereas, the selection of *H. dromedarii* and *H. excavatum* for molecular studies is based on two reasons: (1) They have an economic important more than other tick species found on camels, wherever, *H. dromedarii* is the most dominant tick species, and *H. excavatum* has a wide range of animal hosts, and it can spread the disease from animal host to another through farm animals, and (2) the larval stages of these two tick species are very close to each other when they examined by scanning electron microscope by Abdel-Shafy [43]. The third step of this study is a trial to answer the following question using SDS-PAGE and western blot techniques. “Is it possible to identify an animal infested by *H. dromedarii* or *H. excavatum* serologically?”

Diagnosis of Q fever in camels in most previous publications was conducted by various serological methods. These studies indicated that the camels are a major reservoir of *C. burnetii*. The seroprevalence for Q fever revealed infection rates 7.9, 73, 19, 51.64, 28.26, and 18.6% among camels investigated in United Arab Emirates, Chad, Spain, Saudi Arabia, Iran, and Kenya, respectively [61-66]. In Egypt, antibodies of *C. burnetii* was found in 13% and 70% of camel sera tested by ELISA [14,67] and indirect immunofluorescence assay [68], respectively. Although molecular methods are more sensitive and specific for the diagnosis of the disease, little researches used these methods in the screening of Q fever in either camels or ticks. In general, the percentage of *C. burnetii* infection in camel blood or tick tissues detected by PCR was mostly lower than recorded by serological methods. The infection rate of Q fever detected in camels by PCR was 15.85 % and 10.76% in Saudi Arabia and Iran, respectively [64,69]. In Spain, *C. burnetii* DNA was detected in 3.4 and 6.1% of ticks collected from animals at two different locations [70,71]. In Egypt, the present study is considered the first for molecular characterization of Q fever on either camels or ticks by PCR targeting the IS30A spacer. The results of this study revealed that 52 of 113 blood samples camels (46%) were positive for Q fever infection. This percentage is higher than that recorded before in Saudi Arabia and Iran [64,69]. This may be due to the differences in environmental conditions between Egypt and these two geographical locations or the differences in the sensitivity of the genes used in PCR.

Furthermore, the results of the current study revealed that 10 (5.3%) adult ticks of *H. dromedarii* were infected with *C. burnetii* using PCR. This low Q fever infection agrees with that recorded before in ticks collected from animals in Spain [70,71]. The band size of IS30A spacer of *C. burnetii* was 200 bp in the positive samples of camel blood and tick tissues. The PCR products of *C. burnetii* DNA in camels failed to give sequences while in *H. dromedarii* ticks revealed good sequences with a similarity of 99% with that previously recorded in GenBank. This finding may attribute to the ticks had high *C. burnetii* DNA in comparing with a low density of DNA in the blood.

Meanwhile, the previous study indicated that Q fever was lower in blood and milk, but it is higher in faces and urine [64,72]. Then, this study confirms the presence of *C. burnetii* in *H. dromedarii* which can transmit from infected animals to healthy animals or humans directly by exposure to the infected ticks or by their offspring [73]. In contrary with Altay Çapin *et al*. [74] who found *C. burnetii* in *H. excavatum*, this tick species was found free from Q fever infection in the present study. This finding may attribute to the low number of *H. excavatum* ticks examined by PCR herein.

The taxonomists of ticks could easily identify tick species spread in their country, but they have misidentification rates when they identify tick species imported from other countries [75]. Nowadays, the molecular approach becomes an important trend in the taxonomic of tick species to resolve the misidentification problems. The most DNA markers used for molecular characterization of *Hyalomma* spp. are 18S rRNA, second internal transcript spacer, 12S rDNA, *CO1*, 16S rDNA, and calreticulin [25,76-78]. In the present study, 16S rRNA and *CO1* genes were chosen for molecular characterization of *H. dromedarii* and *H. excavatum* by PCR and sequence analysis based on the report of Lv *et al*. [46] who stated that 16S rRNA and *CO1* genes are trustworthy in distinguishing species of ticks, while 18S rRNA can distinguish ticks at the genera level. The results of this study showed that the PCR products of 16S rRNA and *CO1* genes revealed bands around 440 and 850 bp in length, respectively, for both *H. dromedarii* and *H. excavatum*. Then, the sequencing is imperative to discriminate the two tick species. The gene 16S rRNA gave a good sequencing for *H. dromedarii* with 99% similarity with that recorded in GenBank. This sequence was recorded in GenBank and took number MG757400. However, the PCR products of 16S rRNA gene obtained from *H. excavatum* failed to give a good sequence despite it was repeated for many trials. This finding may be due to the 16S rRNA gene is not appropriate for *H. excavatum* tick, or there was a technical error occurred in preparing the sample for sequencing.

Furthermore, the present study added that the phylogenetic trees of 16S rRNA and *CO1* genes for *H. dromedarii* confirmed that these species locate in the same glade with other *H. dromedarii* previously recorded in GenBank as well as the phylogenetic tree of *CO1* gene for *H. excavatum* was also in a separate glade with others *H. excavatum* previously recorded in GenBank. These findings agree with the previous results that reported by Sands *et al*. [77] and Hekimoglu and Ozer [76] who found that each of *H. dromedarii* and *H. excavatum* locates in a separate clade when they analyzed phylogenetically. Then, this study confirms that the *CO1* gene is used for discrimination between *H. dromedarii* and *H. excavatum*, while 16S rRNA gene is suitable for *H. dromedarii*, but it needs additional trials for *H. excavatum* at least with the Egyptian strain of *H. excavatum*.

SDS-PAGE revealed seven bands for the adults of either *H. dromedarii* or *H. excavatum* with different molecular weights in each. Despite the protein profile in each tick species is different; we cannot depend on the protein profile alone in discriminating these tick species. Overlapping may occur between molecular weights of tick species in case of comparing the protein profiles of a large number of tick species. In western blot analyses, the sera obtained from either infested camel by *H. dromedarii* or infested cattle by *H. excavatum* revealed the same immunogenic bands (100.7, 49.7, 43.9, and 39.6 kDa) against *H. dromedarii*. However, the camel sera exhibited two immunogenic bands (117 and 61.4 kDa) against *H. excavatum*. Furthermore, the sera collected from cattle infested by *H. excavatum* revealed three immunogenic bands (61.4, 47.3, and 35 kDa) against *H. excavatum*. It is thought that the cattle were infested by *H. dromedarii* because the cattle sera revealed the same immunogenic bands that recorded with camel sera. The difference between immunogenic bands for camel and cattle sera against *H. excavatum* may attribute to the camel was infested by other ixodid tick species besides *H. dromedarii* and stimulate the two immunogenic bands. The presence of the three immunogenic bands at 61.4, 47.3, and 35 kDa in reaction of cattle sera with *H. excavatum* and their absence in reaction of camel sera against this tick species indicate to *H. excavatum* infestation in cattle and free *H. excavatum* infestation in camel. This means that it is possible to identify tick species through a serum obtained from an infested animal that has been infested by one tick species. To apply this conception, the sera must be obtained from an animal that has been infested experimentally to avoid cross-reactivity with other tick species in the natural field.

## Conclusion

Molecular analyses indicated that both camels and ticks could be sources for the infection of animals and humans with Q fever. Furthermore, molecular analyses are more accurate tools for discriminating *H. dromedarii* and *H. excavatum* than immunological tools.

## Authors’ Contributions

HHAMA, EEE, SA, HAAA, and EHA participated in the design of the study. HHAMA and SA shared in collecting ticks and blood samples from camels. HHAMA, EEE, and SA participated in conducting PCR, sequences, and phylogenetic trees. EEE and EHA participated in antigen preparation and conducting SDS-PAGE and western blotting techniques. SA wrote the first draft of the manuscript. All authors reviewed and approved the final manuscript.
